# Prevalence of generative artificial intelligence sexualized image usage by adolescents in the United States

**DOI:** 10.1371/journal.pone.0342824

**Published:** 2026-03-18

**Authors:** Chad M. S. Steel

**Affiliations:** Department of Electrical and Computer Engineering, George Mason University, Fairfax, Virginia, United States of America; Indiana University School of Medicine, UNITED STATES OF AMERICA

## Abstract

The prevalence of generative artificial intelligence (GenAI) usage related to sexualized images amongst adolescents is a critical emerging research area. In this exploratory study, a nationally representative online survey of 557 English-speaking individuals aged 13–17 was conducted. Participants were asked about their consensual and non-consensual usage and interactions with sexualized GenAI images. The survey included questions on images created with nudification software as well as content creation software, and asked participants about their creation, sharing, and viewing of this content, as well as that of their peers. Use of nudification tools was widespread, with 55.3% (n = 308) of participants reporting having created and 54.4% (n = 303) having received at least one image. Reported victimization levels of participants was substantial, with 36.3% (n = 202) of individuals reporting having a non-consensual image created and 33.2% (n = 185) of individuals having had at least one image non-consensually shared. Usage was similar across demographic categories, though male participants had higher degrees of regular GenAI sexual image creation and distribution, both consensual and non-consensual. Policymakers need to consider the extensive usage and its normalization and consider educational interventions, and practitioners need to be aware of the high degree and nature of victimization occurring.

## Introduction

Recent advances in generative artificial intelligence (GenAI) have ushered in a paradigm shift in image generation and manipulation. Tools such as Stable Diffusion, DALL-E and Midjourney allow users to create images from text prompts, leveraging large language models (LLMs), through web-based services. This expands artistic possibilities and allows individuals to express their creativity in new and imaginative ways [1,2]. Similarly, locally installed applications on smartphones and computers allow users to create their own visual content and modify existing visual content (for example, placing real individuals into generated or existing photographs seamlessly) using techniques such as inpainting [3]. These techniques have been applied to many legitimate, novel uses, ranging from artificially aging photographs for forensic purposes [4] to enabling smartphone applications that allow individuals to virtually “try on” clothing before buying it [5].

As with many new technologies, GenAI can also be used for maladaptive purposes. For child sexual exploitation material (CSEM) offending, there are new affordances available using these tools. Some of these affordances involve the creation of toolsets (e.g., training models specifically with CSEM content), but the key uses for most individuals engaged with GenAI for CSEM offending mirror the legitimate uses of the technology. These include the generation of CSEM content directly using text prompts and common diffusion models [6], the use of nudification tools to visualize what individuals might look like without clothing [7,8], and the alteration of adult sexual exploitation material to include the faces of minors [9].

One area of particular concern is the increase in the creation and distribution of self-generated sexualized images by minors. These images are now a substantial portion of the images found in CSEM reports, with the Internet Watch Foundation (IWF) reporting that 44% of CSEM shared online was self-produced [10]. Initial estimates of sexting amongst adolescents in the United States found 2.5% created (or had created) sexualized images of themselves, and 7.1% received these images [11]. More recent work identified higher rates of creation and receipt of these images, with usage increasing over time [12], and a meta-analysis found creation and receipt rates of 14.8% and 27.4%, respectively [13]. Additionally, the creation of these images has shown some normalization within the attitudes of adolescents towards these activities [14], and they have become a routine part of adolescent sexual exploration. The IWF reporting at the time and the more recent statistics on sexting, however, did not differentiate between GenAI and non-GenAI images, due both to methodological (the protocols did not look for GenAI images) and technological (the difficulty in differentiating GenAI from other images) reasons.

In addition to the increase in self-generated, sexual images through sexting, the adoption of GenAI technologies by adolescents is expected to continue to grow. In November 2024, Ofcom reported that 54% of British children aged 8–15 had used GenAI in the past year [15]. Smartphone applications that utilize augmented reality (using similar technology to nudification apps) for the purposes of trying on virtual clothing are also more accepted by younger users [16]. With nudification apps becoming easier to find and use [17], familiarization with and normalization from the usage of similar GenAI applications may increase adoption amongst adolescents.

Adolescents are already exposed to sexualized GenAI images in the form of deep fakes. In 2024, 14% of British adolescents under 16 had come across a sexualized image or video created with GenAI in the prior year over social media, video sharing platforms, or email [15]. Deep fakes in general have been found to be met with negative sentiments [18], but age-related differences have not been well studied. Teens in particular may exhibit different behaviors in the creation and distribution of sexualized GenAI images, consistent with the normalization of self-generated sexually explicit images [14].

In addition to general exposure, the harms of deep fakes on victims are just starting to be explored. Victims of GenAI-based sexual exploitation reported issues consistent with other forms of CSEM victimization, including fears of who may have seen the images when in public (hypervigilance) and general avoidance of social media usage, as well as a sense of powerlessness to prevent it and general dehumanization, resulting in permanent life disruptions [19,20]. Limitations based on current laws further constrain the resources available to victims of malicious deep fakes, particularly those of a sexualized nature [21].

The production, viewing, and distribution of pornographic GenAI images of individuals under 18 is illegal in the United States under federal law (18 U.S.Code § 1466A), and does not require that a real individual be depicted [22]. In both the United States and most of Europe, there are gaps in the current laws related to GenAI CSEM, including the legality surrounding the possession and training of tools to produce content [23]. Additionally, while there are no safe harbor provisions in United States law for consensually produced adolescent content, in practice prosecutorial discretion is used to allow for education and administrative remedies in lieu of criminal charges. A full review of the legal issues surrounding GenAI CSEM can be found at [24].

While there has been an increase in individual reports of GenAI misuse by adolescents [25–27], overall prevalence rates are unknown. This research is the first large-scale exploratory effort to measure the usage of GenAI tools to create sexualized images, including nudification tools and image creation tools (based on text prompts or inpainting of existing images), by adolescents. Both consensual and non-consensual use cases are evaluated, including creation and distribution by both participants and by their peers, using an anonymous, online survey.

## Materials and Methods

This study consisted of an anonymous, cross-sectional, Internet-based survey targeting adolescents and their interaction with GenAI sexual imagery.

### Participants and setting

This research obtained data through an online, anonymous survey hosted by Qualtrics. The survey consisted of multiple questions related to the interactions of participants with sexualized images created using GenAI. The population of the survey was English-speaking adolescent teens living in the United States between the ages of 13 and 17, inclusive. A target sample of 500 individuals was solicited, based on the ability to identify a medium effect size (.3) for in-group demography variations, between January 11 and January 24, 2025. A pilot distribution of 50 responses was solicited prior to the full launch to ensure survey flow, response controls, and timing were performing as expected. The researchers had no direct involvement in the selection of participants, who were drawn by the providers from a group of individuals whose parents pre-identified them as potential candidates as part of a panel service offered by Qualtrics. The sample was designed to be representative and used a non-probability quota-based methodology based on demographic data previously provided by the participants [28]. No direct compensation was provided to participants by the research team, however Qualtrics provides compensation to panel members ranging from gift cards to airline miles based on proprietary algorithms.

Due to the age of the participants, a two-stage process of informed consent was utilized. First, the parents of potential participants were provided a link to take a survey by Qualtrics. Those parents who clicked the link were provided details on the survey’s nature, including a copy of the questions as well as a detailed consent form outlining the risks and benefits of the study. The information provided to the parents confirmed the results of the study would be anonymous and the anonymized data available to future institutional review board approved studies, and that they would not have access to their child’s responses. Parents were requested to allow their children privacy to fill out the questions but encouraged to discuss the questions and the topic with their children afterward. Of the 9098 parents who were presented the form, 967 agreed to their child’s participation. The children of parents who provided consent were then presented with a detailed assent form containing age-appropriate language and detailing the same information as the consent form. Of the 967 adolescents presented the form, 936 agreed to continue with the survey. Following their completion of the survey, adolescents were encouraged to voluntarily discuss the issues addressed in the survey with their parents.

The surveys were anonymous, and no identifying information, including network information (e.g., IP addresses), was retained. The parents were provided an anonymous link to the consent, and, if they chose to consent, their child was provided a separate, unrelated anonymous link to the survey. The specific demographic information collected was limited to avoid unintentionally allowing ex post facto identification of a specific participant through aggregate demographic details (e.g., no zip codes were collected). The survey was additionally designed to avoid any free-text entry boxes through which participants may inadvertently self-identify. Due to the anonymous nature of the study, no direct debriefings were possible, but links to counseling resources were provided to both the participants and their parents if they wanted to discuss any reported victimization or the topic area in general. Additionally, law enforcement contacts were provided to both participants and parents for voluntary reporting of any related events. Finally, contacts were provided for both the research team and the institutional review board to both parents and participants should they have any detailed questions or concerns about the research.

The adolescents were permitted to cease their participation in the survey at any point up until submission but following submission their data was no longer identifiable, and only anonymized data was available to the research team.

There were multiple levels of quality assurance built into the process. Qualtrics used browser fingerprinting to avoid duplicate entries, anti-botnet features such as CAPTCHAs, and IP geolocation to ensure respondents were within the United States. They additionally included a timing check, and any responses below half the median time were discarded as “speeders”. Finally, they included straight-line and Christmas-tree checks (any that were consistently answered across three separate matrices) to ensure answer integrity [29]. In addition to integrity checks built-in by Qualtrics, two additional checks were provided by the researchers. First, a question confirming the ages of the participants was asked. Those identifying an age outside of the 13–17-year-old range were excluded from continuing with the survey. Second, an attention check was built into the survey to improve response quality. A total of 115 participants failed to complete the survey and integrity checks, and 264 were identified as outside of the target age group, resulting in n = 557 total surveys available for analysis.

### Questionnaire

Demographic questions on race, gender (participants were asked how they describe themselves), sexual orientation, region and age were collected from all participants. For the purposes of this study sexualized GenAI images were operationally defined as naked, still images or videos, either of their peers (“other individuals between the **ages of 13 and 17** years old”) or of adults (“any other individuals who are 18 years of age or older”). The primary questions were related to participants’ interactions with relevant content, and specific questions were asked about both consensual and non-consensual creation and distribution of naked GenAI images. Questions were focused on four areas:

- Use of nudification tools by participants
- Use of nudification tools by others in their peer group
- Use of general GenAI image creation/alteration tools by participants
- Use of general GenAI image creation/alteration tools by others in their peer group

Participants were asked about their own creation of images (of themselves or others), their distribution of images, and their receipt of naked images of both adults and their peers. For each question, a five-point, custom Likert scale was used with the following values:

- I have never done this
- I have only done this once or twice
- I have done this infrequently
- I have done this frequently
- I have done this on a regular basis

The questions were specifically worded based on prior research questions related to CSEM activities to minimize the impact of social desirability bias on responses [30].

Participants were provided age-appropriate, plain text operational definitions and directions for each of the sections. For example, nudification tools were defined as follows:

The following questions are about the use of nudification tools. These are smartphone apps, websites, or other software that shows what individuals might look like without clothing.

AI image creation tools were operationally defined as those that “use text prompts or existing images as inputs… [creating] new images from scratch or using existing images and altering them with AI” to create naked content.

Finally, respondents were asked to confirm if they filled out the questions in private, with a parent present, or with another individual present to allow researchers to identify any potential observer influence on responses.

### Analysis

The results were collected and stored on the university file share, and all analysis was performed in R (4.5) using R Studio. Wilcoxon rank-sum tests for two category comparisons and chi-squared tests for multi-category comparisons were utilized for the ordinal and categorical comparisons, and Spearman correlation for age comparisons. An additional chi-squared test was performed for age comparisons as well to identify potential non-linear effects. A statistical significance level of.01 was used where p values were reported, with Bonferroni corrections applied.

### Ethics

The study design and protocols, including an analysis of the potential risks and benefits, was approved by the George Mason University Institutional Review Board on 19 December 2024.

## Results

The research sample consisted of a representative, demographically diverse group of English-speaking, United States teens. The survey was completed by 557 individuals (51.0% male, 48.3% female, 0.5% non-binary, 0.2% not specified; 73.2% Caucasian, 15.6% African American, 2.3% Asian, 1.4% Native American, 4.7% with two or more races; 89.2% identified as heterosexual, 6.5% as bisexual, and 3.2% as homosexual). With respect to privacy when taking the survey, 70.6% reported taking it alone, 20% with a parent present, 0.5% with another individual present, and 9% chose not to answer. Table 1 shows the overall sample characteristics.

**Table 1:** Sample Demographics.

Characteristic	n	Percentage	95% CI
*Age*			
13	97	17.4%	(14.3 - 20.6)
14	92	16.5%	(13.4 - 19.6)
15	109	19.6%	(16.3 - 22.9)
16	105	18.9%	(15.6 - 22.1)
17	154	27.6%	(23.9 - 31.4)
*Race*			
American Indian/Native American or Alaska Native	8	1.4%	(0.4 - 2.4)
Asian	13	2.3%	(1.1 - 3.6)
Black or African American	87	15.6%	(12.6 - 18.6)
Multiple Races	26	4.7%	(2.9 - 6.4)
Native Hawaiian or Other Pacific Islander	2	0.4%	(−0.1 - 0.9)
Other	12	2.2%	(0.9 - 3.4)
Prefer not to say	1	0.2%	(−0.2 - 0.5)
White or Caucasian	408	73.2%	(69.6 - 76.9)
*Spanish, Hispanic, or Latino Origin*			
Yes	94	16.9%	(13.8 - 20)
No	463	83.1%	(80 - 86.2)
*Gender*			
Female	269	48.3%	(44.1 - 52.4)
Male	284	51.0%	(46.8 - 55.1)
Non-binary/ third gender	3	0.5%	(−0.1 - 1.1)
Prefer not to say	1	0.2%	(−0.2 - 0.5)
*Sexual Orientation*			
Bisexual	36	6.5%	(4.4 - 8.5)
Heterosexual	497	89.2%	(86.7 - 91.8)
Homosexual	18	3.2%	(1.8 - 4.7)
Other	1	0.2%	(−0.2 - 0.5)
Prefer not to say	5	0.9%	(0.1 - 1.7)
*Region*			
Midwest	125	22.4%	(19 - 25.9)
Northeast	102	18.3%	(15.1 - 21.5)
South	145	26.0%	(22.4 - 29.7)
West	181	32.5%	(28.6 - 36.4)
Other	4	0.7%	(0 - 1.4)

Overall, usage of GenAI tools to create naked images was widespread. Self-generation of nudified images (use of a nudification tool to create an image of oneself) was the highest modality, with 55% of participants having done this at least once, 44% having shared a self-image created with nudification tools, and with 54% of recipients having received a self-generated image from a nudification tool. Content creation and sharing appeared to be generally focused on peer group images and not adult images, with only 35% having created and 33% having shared naked images of adults. Additionally, the usage of nudification tools was significantly higher than the usage of GenAI creation tools (z = −3.66, p < .001) comparing any nudification image interaction with any traditional GenAI image interaction, indicating a higher impact of victimization (for the purposes of this research, creation or distribution of images without their permission) as nudification tools require an actual photo of a real individual as a basis image. Overall details by activity are available in the online supplemental material (S1 Table).

Only male and female gender identities were analyzed for differences as insufficient numbers of non-binary gender identities were present in the sample to evaluate them effectively. There was an overall similar usage between genders, but seven categories of usage had higher usage by males, including the creation of peer images with nudification tools both with (W = 31967, p < .001) and without (W = 32592, p < .001) their permission; having received re-distributed images of their peers (W = 31953, p < .001); using image creation tools to create images of adults (W = 32521, p < .001); sharing naked images of themselves (W = 31588, p < .001) and of others in their peer group (W = 32496, p < .001) created with image creation tools; and having had images of adults (W = 32278, p < .001) and other peers (W = 30863, p < .001) shared with them by their peer group (S2 Table).

There were no statistically significant differences in usage based on age across any of the activities, either through a linear fit based on age or categorically (S3 Table). When comparing prevalence rates across sexual orientation, only sharing of AI-generated images of adults differed significantly across orientations, *X*^*2*^(16)=42.47, p < .001 (S4 Table). A post-hoc analysis of the results with Bonferroni correction identified individuals who identified as “Other” having a significantly higher residual (4.823, p < .001) (S5 Table). No statically significant prevalence differences were identified associated with race (S6 Table).

No statistically significant differences were identified based on the presence of a parent or other individual when taking the survey in the responses (S7 Table).

## Discussion

Better education is needed on the healthy and safe usage of GenAI technology, which has been previously believed to disproportionately impact women [20] and was borne out in this research, though both genders were found to be substantially impacted in this study as both users and victims. While only a few questions showed statistically significant distributions by gender, both self and peer creation and distribution were higher for males in terms of regular use and for having experimented (engaged at least one time) with the technologies. The lack of significant gender differences with a large effect size for most of the actions asked about is consistent with Madigan et al.’s work on youth sexting, and may be a continuation of that trend reflecting technological advances [13]. The one differentiator related to sexual orientation was a higher incidence of “Never” within those who identified as “Other”. This may be due to individuals who self-identify as asexual being included in this category, but additional research with a larger dataset and more detailed categorization is needed to verify this hypothesis.

Education interventions additionally need to be multimodal and need to occur at a younjg enough age to address the issue before it occurs [31]. Since there was no statistically significant age difference in either usage or victimization across the age groups (though as with gender, smaller effect sizes may be identified with higher powered studies), the education should occur prior to age 13 to be most impactful. Prior work on rape myth acceptance, particularly that targeting bystanders (in this context, individuals receiving or being shown unsolicited images) and how they should respond, as well as impact education to prevent perpetration are warranted and can potentially inform effective approaches [32].

In terms of victimization, the usage of and sharing of content from nudification tools was higher than that of general GenAI creation tools, portending a higher degree of direct victimization as nudification tools generally involve a known/direct child victim. These results represent a lower bound in victimization statistics, as perpetration by adults (use of these tools on minors or sending images to minors) was not incorporated into this study. Secondary victimization through the further distribution of images without consent is likewise a lower bound as participants may not know the extent of onward transmission. Further research, particularly longitudinal research, on the long-term impacts of creating sexualized images (particularly self-images) using these tools is needed.

Most of the activities surveyed represent the creation of CSEM, a violation of federal law in the United States. Because many of the actions were consensual and involving peers, policymakers need to consider if these activities, which may be considered part of normal sexual exploration, warrant specific legal exceptions beyond current discretionary prosecution. Specifically, carve-outs for consensual generation and sending between individuals in the same age group warrant consideration. Better controls on the usage of nudification and other GenAI applications to detect CSEM production is also needed. While calling for application makers to provide better controls to limit their use by minors is necessary, many of the applications are gray market tools and age restriction controls are not likely to be fully successful [17] unless coupled with an education strategy.

The long-term impacts on CSEM distribution additionally need to be considered in light of these findings, assuming they remain stable over time. GenAI detection tools need to specifically target modified images to ensure victim identification given the significant use of nudification applications. Finally, the prevalence of GenAI nudification and creation tool usage by adults, both legally and illegally (to produce CSEM or non-consensual explicit images) needs to be studied [33].

## Limitations

This research was an exploratory study conducted on English-speaking teens 13–17 years of age within the United States, and additional work would be required for generalizability to other populations. Because of the relatively recent introduction of the latest generation GenAI technologies, these results only represent a point-in-time analysis and future research once the technologies mature, as well as longitudinal research, are needed before drawing any broad conclusions. Although controls were put in place both by Qualtrics and the research team to obtain accurate survey results through attention checks, timing issues, and verification of privacy, there are general issues with Internet surveys that will always be present in this type of research. Specific to this research, the IRB-approved protocol provided parents the ability to review the full questionnaire before allowing their children to participate. This introduces a potential selection bias, where parents who opt out of participation for social, religious, cultural, or other reasons may have adolescents that differ in their GenAI usage from those whose parents opted in. The number of respondents was selected to have sufficient power to perform larger subgroup analyses but was not sufficient to draw conclusions about smaller subgroups (e.g., non-binary teens) or to identify small effect sizes. Participants were asked about their use of GenAI and interactions with sexualized images of others in their peer group and of adults, however no questions were asked of their use of these tools to create images of those younger than 13, so the numbers represent an upper bound of individuals in this group using GenAI to interact with CSEM imagery. Finally, the questions asked about the generation and sharing of naked images – other sexualized imagery with no nudity is possible, and some naked images may not be sexualized, however this language was chosen for ease of understanding and interpretation.

## Conclusions

A significant percentage of adolescents in this national survey were found to engage in the use of GenAI applications to create sexualized images, including nudification applications. This represents a potentially significant source of CSEM creation and distribution, and shows that, to some degree, risky engagement with GenAI is widespread in this population. The normalization of these activities, and the harm to victims for non-consensual creation and/or distribution needs further study for its impact on prevention, treatment and deterrence efforts. While the usage was widespread across demographic categories, most of the usage appears to have been exploratory rather than habitual based on the frequencies reported, providing an opportunity for positive intervention. Because there were no age-related differences in GenAI usage, age-appropriate education-based intervention efforts on the risks associated with AI tools and sharing of images need to start prior to the age of 13. Additional training for law enforcement on handling both offenders and victims, as well as digital forensics specialists on the identification of GenAI images, is needed. Finally, decision makers need to consider the prevalence and nature of these use cases in developing new legislation related to GenAI CSEM.

## Supporting information

S1 Table Descriptive Statistics of Individual AI Activities. (XLSX)

S2 Table Gender Differences in Sexualized Generative AI Usage. (XLSX)

S3 Table Age Differences in Sexualized Generative AI Usage. (XLSX)

S4 Table Summary of Sexual Orientation-Related Differences in Sexualized Generative AI Usage. (XLSX)

S5 Table Post-hoc Analysis of Sexual Orientation. (XLSX)

S6 Table Summary of Race-related Differences in Sexualized Generative AI Usage. (XLSX)

S7 Table Summary of the Impact of Other Individuals Presence During Survey Taking. (XLSX)
